# Mineral trioxide aggregate affects cell viability and induces apoptosis of stem cells from human exfoliated deciduous teeth

**DOI:** 10.1186/s40360-018-0214-5

**Published:** 2018-05-15

**Authors:** Chia-Ling Tsai, Mu-Chan Ke, Yi-Hao Chen, Hsi-Kung Kuo, Hun-Ju Yu, Chueh-Tan Chen, Ya-Chi Tseng, Pei-Chin Chuang, Pei-Chang Wu

**Affiliations:** 1 Department of Dentistry, Kaohsiung Chang Gung Memorial Hospital and Chang Gung University College of Medicine, Kaohsiung, Taiwan; 2 Department of Ophthalmology, Kaohsiung Chang Gung Memorial Hospital and Chang Gung University College of Medicine, 123, Da-Pi Road, Niao-Sung District, Kaohsiung, 88301 Taiwan, Republic of China; 3 Department of Medical Research, Kaohsiung Chang Gung Memorial Hospital, and Chang Gung University College of Medicine, Kaohsiung, Taiwan; 4 Center for Translational Research in Biomedical Sciences, Kaohsiung Chang Gung Memorial Hospital, Kaohsiung, Taiwan

**Keywords:** Stem cells, Human exfoliated deciduous teeth, Apoptosis, Cytotoxicity, Mineral trioxide aggregate

## Abstract

**Background:**

Mineral trioxide aggregate (MTA) is widely used for pulp-capping procedures in permanent teeth and as a gold standard material in endodontics. The aim of the study was to investigate the effect of MTA on cell viability and apoptosis when MTA is directly in contact with Stem Cells from Human Exfoliated Deciduous Teeth (SHEDs).

**Methods:**

MTA was mixed and coated in the bottom of a 24-well plate. SHEDs collected and cultured from normal exfoliated human deciduous teeth (passages 3–4) were seeded on square cover glasses. The glasses with seeded SHEDs were incubated in the plates with or without MTA coating. They were divided into four groups: MTA direct contact, direct control, MTA indirect contact, and indirect control. After 1, 2 and 3 days of culturing, cell morphology was observed and cell viability was assessed by the WST-1 cell cytotoxicity assay. TUNEL assay, immunofluorescent labeling and western blot analysis were used to study the effects of MTA on SHEDs apoptosis.

**Results:**

MTA impaired cell viability of SHEDs in 1, 2 and 3 days, and the effect of direct contact was more severe. Cell apoptosis with positive Annexin V and TUNEL staining was noted when there was direct contact with MTA. Western blot analysis revealed that Bcl-2 and Bcl-xL decreased after SHEDs were in contact with MTA.

**Conclusions:**

This study shows that direct contact with 1 week post-set MTA significantly decreases the viability of SHEDs and induced cell apoptosis. The results suggest that there is a possible cytotoxic effect of pulp tissue when there is direct contact with MTA. Different responses would be expected due to the strong alkaline characteristics of fresh mixed MTA.

## Background

Dental pulp capping is indicated for teeth that have had pulp exposure. It can offer an alternative to root canal therapy when pulp is exposed with reversible injury or without signs of inflammation, thereby offering a more conservative approach. Ultimately, the goal of treating the exposed pulp with an appropriate pulp-capping material is to promote the dentinogenic potential of the pulpal cells. Mineral trioxide aggregate (MTA) is widely used for pulp-capping procedures in permanent teeth and as a gold standard material in endodontics [1]. It has been investigated for endodontic applications since the early 1990s and became commercially available as ProRoot MTA (Tulsa Dental Products, Tulsa, USA) in 1998. MTA was broadly used in endodontics for various applications such as root-end filling, root perforation and reabsorption repair, apexification, pulp capping and dressing for pulpotomy in primary and permanent teeth [2]. This widespread implementation is explained by MTA’s beneficial properties, including its antimicrobial action [3], insolubility in oral fluids and radiopacity [4], good sealing ability [5], and especially its biocompatibility [6, 7] and bioactivity [8].

Theoretically, set MTA contains calcium hydroxide in a silicate matrix which is what attributes the high pH to MTA [9]. MTA maintains its high pH throughout a period of more than 2 months [10]. According to a study of the responses of cells to pH changes, when the pH was raised from 7.3 to 8.9, a marked contraction and detachment of cells occurred [11]. It may be inferred that the cells should express similar unfavorability in cell culture with MTA. Diametrically, a number of investigations have shown that MTA is one of the least cytotoxic dental materials by using various cell culture systems [12]. Many biocompatibility studies have been conducted in vitro and have shown favorable biological properties of MTA in terms of absence of cytotoxicity, lack of genotoxicity, lack of reactive oxygen species production [7], promotion of bone cell adhesion [13, 14], and a slight increase in cell proliferation [6, 15, 16]. It has been demonstrated that MTA induces repair and/or regeneration of mineralized tissues in vivo [17]. Osteogenesis has been observed when MTA implants were placed in intraosseous sites in rats, suggesting an osteoconductive behavior of the endodontic cement [18].

The responses of pulp in primary teeth to MTA pulpotomies and pulp capping were also favorable from clinical and radiographic perspectives [19]. However, a variety of histological responses, including normal or irregular odontoblasts, intra- pulpal calcifications, internal resorption, and inflammatory infiltrate or pulp necrosis were noted [20]. More cytological support is necessary for the use of MTA as a pulp capping material in primary teeth [21]. Some of these studies used human dental pulp stem cells from permanent teeth for in vitro assays [22–25], but few of them used dental pulp stem cells from primary teeth [26, 27]. Meanwhile, most of the studies evaluated the materials by culturing cells with diluted eluates from the MTA, which is distinctly different from the clinical application of MTA that was mixed and directly dressed on the exposed pulp tissue.

The dental mesenchymal stem cells (MSCs) play a role in tooth development as well as in tooth homeostasis and repair [28]. There are many kinds of dental MSCs, such as dental pulp stem cells (DPSC), stem cells from exfoliated deciduous teeth (SHED), periodontal ligament stem cells (PDLSC), stem cells from apical papilla (SCAP) and stem cells from dental follicle (DFSC) [29]. The discovery of stem cells from human exfoliated deciduous teeth (SHED) has offered a potentially noninvasive source of dental stem cells [30]. SHEDs are multipotent cells with high differentiation potential, osteogenic and chondrogenic potential, and express pluripotent stem cell markers [31]. They demonstrated differences from DPSCs of permanent teeth in terms of growth, differentiation characteristics, and gene expression profile [32]. Several studies have demonstrated that many growth factors and stress can induce differentiation from dental pulp cells [33–35]. The MTA-induced odontoblastic differentiation of human DPSCs was investigated [36]. However, the detail of the direct interaction of MTA with SHEDs is still not known. In this study, we want to clarify if there was any overlooked cytotoxic and possible apoptosis effect of MTA when in direct contact with SHEDs.

Apoptosis is known as type I programmed cell death. It is a tightly controlled process crucial for tissue homeostasis. Apoptosis can be triggered by extracellular or intracellular stimuli with extrinsic or intrinsic pathway activation [37]. The B cell lymphoma 2 (BCL-2) gene family encodes more than 20 proteins that regulate the intrinsic apoptotic pathway and the balance of cell survival and death [38, 39]. BCL-2, BCL-XL, BCL-W, MCL1 and BCL-2A1 are anti-apoptotic proteins and they inhibit the essential apoptosis effectors including BAK and BAX [40].

The aim of the present study was to investigate the effects of MTA on cell viability and apoptosis when in direct contact with SHEDs.

## Methods

### SHED primary culture and identification

The experimental protocols involving human tissue and cells were approved by the Institute Review Board of Chang Gung Memorial Hospital (#100-4678B). Dental pulp was extracted from normal exfoliated human deciduous teeth of 5 to 7-year-old children (8 patients) under local anesthetics at the Outpatient Department of Pediatric Dentistry. Written informed consent was obtained from the patients’ guardians. The pulp separated from a remnant crown was digested in a solution of 2 mg/ml collagenase type I (Millipore, Temecula, CA) and 4 mg/ml dispase (Gibco, NY, USA) for 1 h at 37 °C. Cells were seeded into 3 cm plates (Corning incorporated, NY, USA) with the culture medium and then incubated at 37 °C in 5% CO_2_ for the primary culture. The culture medium was Minimum Essential Medium Alpha Medium (Gibco, NY, USA) supplemented with 15% FBS (Gibco, NY, USA), 100 μM L-ascorbic acid 2-phosphate (Sigma-Aldrich, MO, USA), 2 mM L-glutamine(Gibco, NY, USA), and 100 units of Antibiotic-Antimycotic (Gibco, NY, USA). The cell colonies that showed containing over 90% CD105, CD90, CD73, CD44 and CD29 surface antigens confirmed by flow cytometry were considered as SHEDs in this study.

### MTA Preparation

Under sterile conditions, 1 g PROROOT MTA was mixed(Dentsply Tulsa dental, TN, USA) with 5 ml dd H_2_O (w/p ratio is 5:1) using a mixing stick for about 1 min to ensure all the powder was hydrated. The bottom of 24-well plates was coated (Corning incorporated, NY, USA) with 400ul creamy mixed MTA in each well. The bottom area of the 24-well plate is 200 mm^2^.

The plates with MTA coating wells were set for 1 week at 37 °C in a humidified 5% CO_2_ and 95% air atmosphere for further cell study.

### SHEDs cultured with MTA

**Fig. 1:**
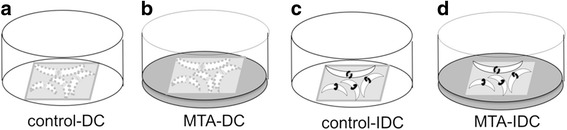
The control groups (**a** and **c**): no MTA coating on the bottom of the wells. The experimental groups (**b** and **d**): MTA coating on the bottom of the wells. The SHEDs cells were seeded on the square cover glasses. In the direct contact (DC) groups (**a** and **b**), the surface of square glass with seeded cells faced downward and toward the bottom of the well. In the indirect contact (IDC) groups (**c** and **d**), the surface of square glass with seeded cells faced upward

To evaluate the reaction of SHEDs when in direct or indirect contact with MTA, SHEDs (passages 3–4) were seeded at an initial density of a 2 × 10^4^ cell/ a piece of 12 mm square cover glass (Deckgläser, lauda-königshofen, Gemany) for 24 h with culture medium (the same medium as used to grow the cells). Then, the cover glasses with SHEDs were moved and incubated with 1 ml fresh culture medium in the plates with or without MTA coating for 1, 2, or 3 days. The SHEDs were divided into four groups: MTA direct contact, direct control, MTA indirect contact, and indirect control. In the MTA direct contact group, the cover glass with SHEDs was inversely placed onto the MTA-coated well and the surface of MTA was in direct contact with the SHEDs. In the direct control group, the cover glass with SHEDs was also inversely placed into the well without MTA coating. In the MTA indirect contact group, the cover glass with SHEDs was placed upright and inside the MTA-coated well and SHEDs were not in contact with the MTA. In the indirect control group, the cover glass with SHEDs was also placed upward into the well without MTA coating (Fig. 1). Each group was studied in triplicate experiments. During the incubation period of 3 days, the cell morphology was examined every day under a phase-contrast microscope (CK40 Culture Microscope; Olympus American Inc., Melville, NY) and photographed.

### Cell viability and proliferation assay

The cell viability and proliferation assay was measured by the Cell Proliferation Reagent WST-1 (Roche, Mannheim, Germany). The cells were cultured with or without MTA for 1, 2, or 3 days, and the Wst-1 assay was performed according to the manufacturer’s protocol. At the end of the culture period, the medium was discarded and the wells, including the square glasses, inside were washed with PBS. Each well was filled with a 1:9 solution of Wst-1 in fresh medium and incubated at 37 °C for 30 min. The solution in each well was transferred to a new 96-well plate. The spectrophotometric absorbance at 450 nm was then measured using an ELISA analyzer. All experiments were performed at least 3 times. Differences between the 2 groups were analyzed using the Student’s t-test.

### In situ cell death detection

To detect the dead cells, the in situ cell death detection kit (Roche,Mannheim, Germany) based on the TdT-mediated dUTP nick end labeling (TUNEL) to detect DNA fragmentation was used. The cells that were cultured in 1 U/ul of DNase I (Zymo Research, CA, USA) for 4 h at room temperature were used as a positive control. In the permeabilization step, samples were treated with 0.1% Triton X-100 (Sigma-Aldrich, MO, USA) in 0.1% sodium citrate (Sigma-Aldrich, MO, USA) for 10 min at 20 °C. After the TUNEL reaction, samples were counterstained with DAPI and then directly analyzed using fluorescence microscopy (Leica DMI3000B, Wetzlar, Germany).

### Detection of apoptosis and/or necrosis by Annexin V/7-AAD staining

To detect apoptosis and/or necrosis, the Dual Detection Reagent (Enzo Life Sciences, NY, USA) containing an apoptosis detection reagent (Annexin VEnzoGold) and a necrosis detection reagent (7-AAD) was used. The cells were fixed with 2% formaldehyde. The cover glasses with cells were washed with diluted phosphate buffered saline and then reacted with 50 mL of Dual Detection Reagent containing the apoptosis detection reagent in 1× binding buffer. The samples were incubated at room temperature for 15 min in the dark. After staining, the cells were washed with binding buffer and covered. The stained cells were observed under a fluorescence microscope (Leica DMI3000B, Wetzlar, Germany) with a filter set for Annexin V-EnzoGold (Ex/Em: 550/570 nm) and 7-AAD (Ex/Em: 546/647 nm).

### Western blot analysis

A 2× Laemmli sample buffer (BIO-RAD, CA, USA) containing 65.8 mM Tris-HCl, pH 6.8, 2.1% SDS, 26.3% (*w*/*v*) glycerol, and 0.01% bromophenol blue was used to extract protein samples which were then loaded onto a 10% sodium dodecyl sulfate-polyacrylamide gel (BIO-RAD, CA, USA). Electrophoresis was performed and proteins were then transferred onto nitrocellulose membranes. The membranes were blocked using 5% skim milk (Sigma-Aldrich, MO, USA) in TBS (Sigma-Aldrich, MO, USA) containing 0.05% Tween-20 (Sigma-Aldrich, MO, USA) for 1 h at room temperature, after which they were incubated overnight at 4 °C with the specific primary antibodies. The anti-GAPDH antibody (Millipore, Temecula, CA) was used to ensure equal protein loading. After incubation, the membranes were washed and incubated with HRP-conjugated secondary antibodies. Chemiluminescence was used to detect the protein bands. Antibodies used in this study included rabbit monoclonal anti-Bcl2 (Cell signaling, MA, USA), anti-Bcl-X_L_ (Cell signaling, MA, USA), rabbit polyclonal anti-Bax (Cell signaling, MA, USA), secondary anti-rabbit IgG HRP-linked antibodies (Cell signaling, MA, USA) and secondary anti-mouse IgG, HRP-linked antibodies (Cell signaling, MA, USA).

## Results

### SHEDs confirmed by flow cytometry

According to the flow cytometry, more than 90% of the 3rd passage SHEDs expressed mesenchymal stem cell markers including CD105, CD90, CD73, CD44 and CD29 on their surfaces. The hematopoietic surface markers CD14 and CD34 only expressed 0.1 and 0.2%.

### Worse cell adhesion in MTA groups

**Fig. 2:**
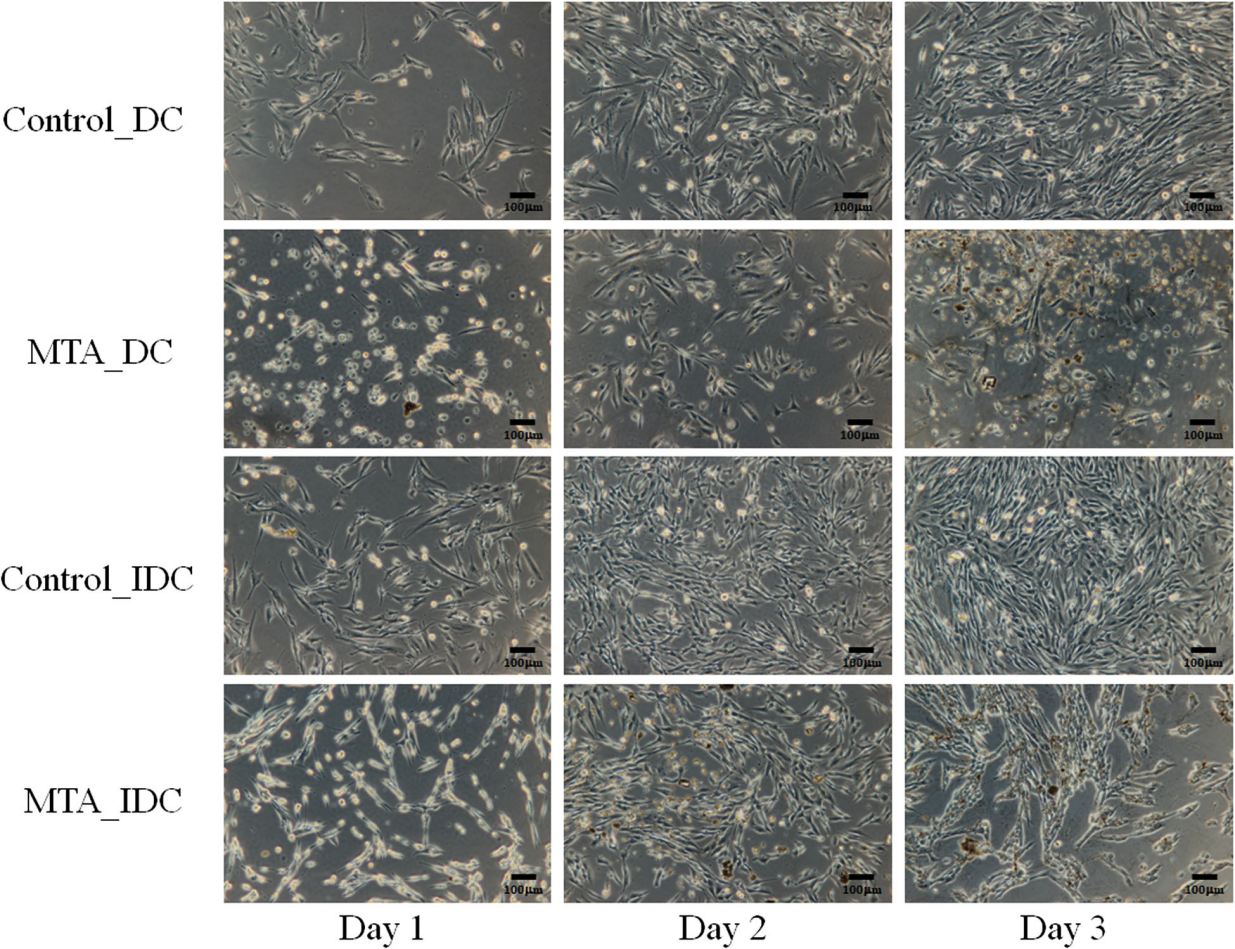
Representative morphology of SHED cells in contact with MTA or not for 1, 2, and 3 days. In the MTA_DC and MTA_IDC groups, sparse shrinkage spindle-shaped cells and worse cell adhesion compared with the control groups were observed. On day 1, many cells detached from the glass slip and floated in the culture medium. The dispersed cells in the groups in which MTA existed showed a poignant contrast to the control. Black bar, 100µm

In the direct and indirect contact groups with MTA, sparse shrinkage spindle-shaped cells and worse cell adhesion compared to the control groups were observed. On day 1, many cells detached from the glass slip and floated in the culture medium. In the first 3 days, the number of attached cells clearly decreased (Fig. 2).

### Direct contact with MTA decreases cell viability

**Fig. 3:**
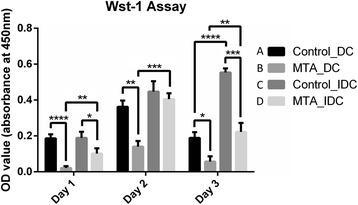
Wst-1 assay was used for cell viability evaluation. Except for the Day 2 IDC pairs, the existence of MTA significantly inhibited cell proliferation compared with the control groups. (A vs. B, C vs. D) When MTA existed, direct or indirect contact also influences cell viability significantly. Comparing B and D in Day 1, 2, or 3, direct contact with MTA significantly inhibited cell proliferation. Data represent mean values (±SD) of triplicate samples per condition. Graphs are representative of results obtained in three independent experiments. * represents for *p* < 0.05, ** for *p* < 0.01, *** for *p* < 0.001 and **** for *p* < 0.0001

The cell viability of SHEDs in contact with MTA was evaluated by the WST-1 assay for 1, 2, or 3 days. The existence of MTA significantly inhibited cell proliferation compared with the control groups. When MTA was present, direct or indirect contact also influenced cell viability significantly. However, the cell viability in the indirect contact groups was not significantly different with or without MTA in day 2 (Fig. 3).

### Identification of DNA fragmentation by fluorescent TUNEL assay

**Fig. 4:**
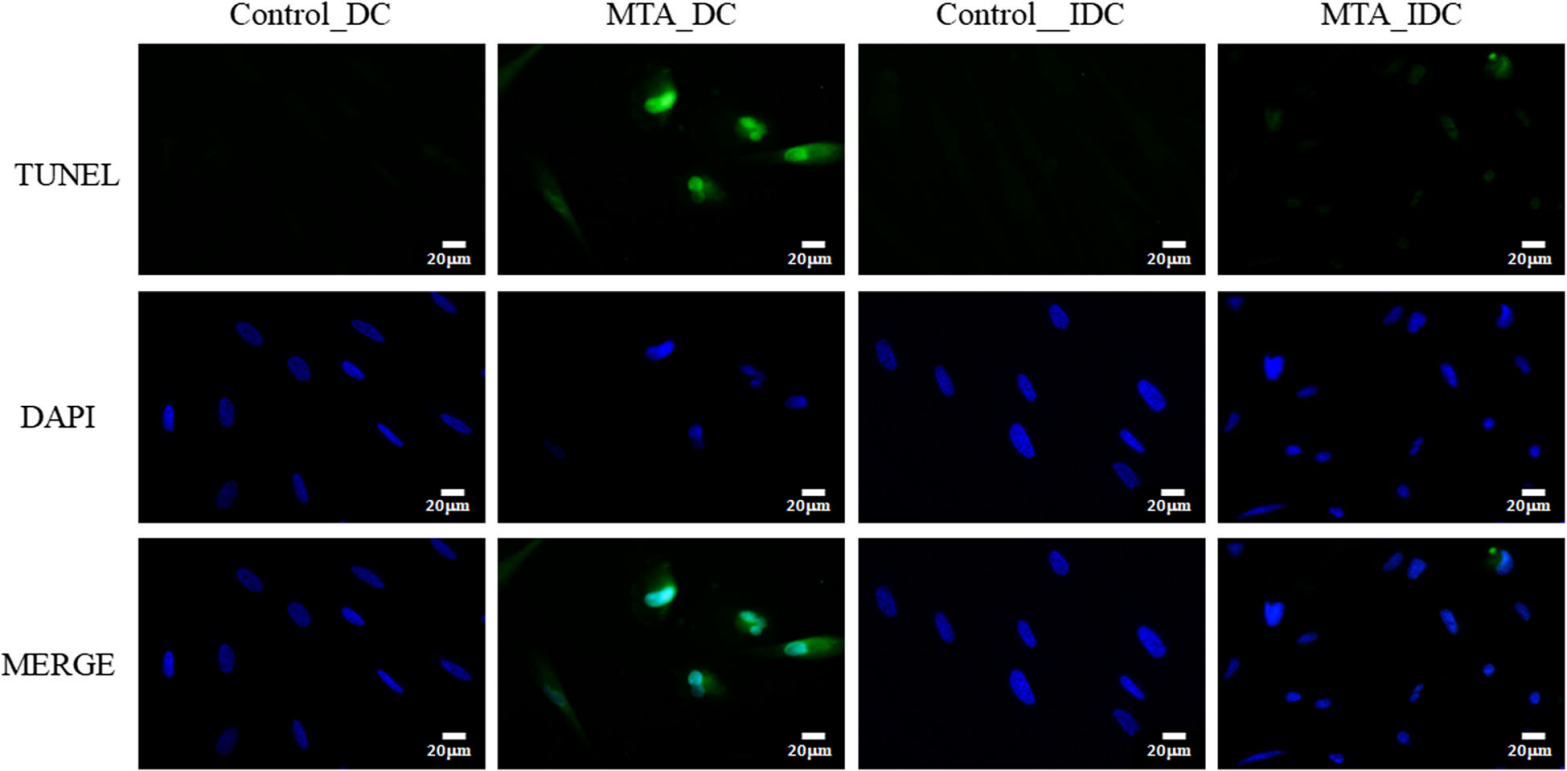
Images of SHED cells with fragmented DNA marked using a TUNEL assay (In situ cell death detection kit, AP, Roche) at 2 days incubated with direct or indirect MTA contact. Positive cells show a bright green nucleus. DAPI staining was used to check the location of the nucleus. Apoptosis was indicated by TUNEL-positive cells. MTA_DC specimens showed positive staining, whereas the MTA_IDC specimen also showed positive staining, but not as obviously. White bar, 20µm

To study the effects of MTA on SHEDs apoptosis, we exposed SHEDs to MTA for 2 days, and performed the TUNEL assay to detect DNA ends. Because apoptosis is characterized by DNA fragmentation, increased staining in the nucleus (TUNEL-positive cells) indicates apoptosis. Negative results were seen in the Control_DC or Control_IDC groups, whereas the MTA_DC group showed positive stains. However, the MTA_IDC group also showed positive stains, but not as obviously (Fig. 4).

### Apoptosis assessment using Annexin V/7-AAD staining assay

**Fig. 5:**
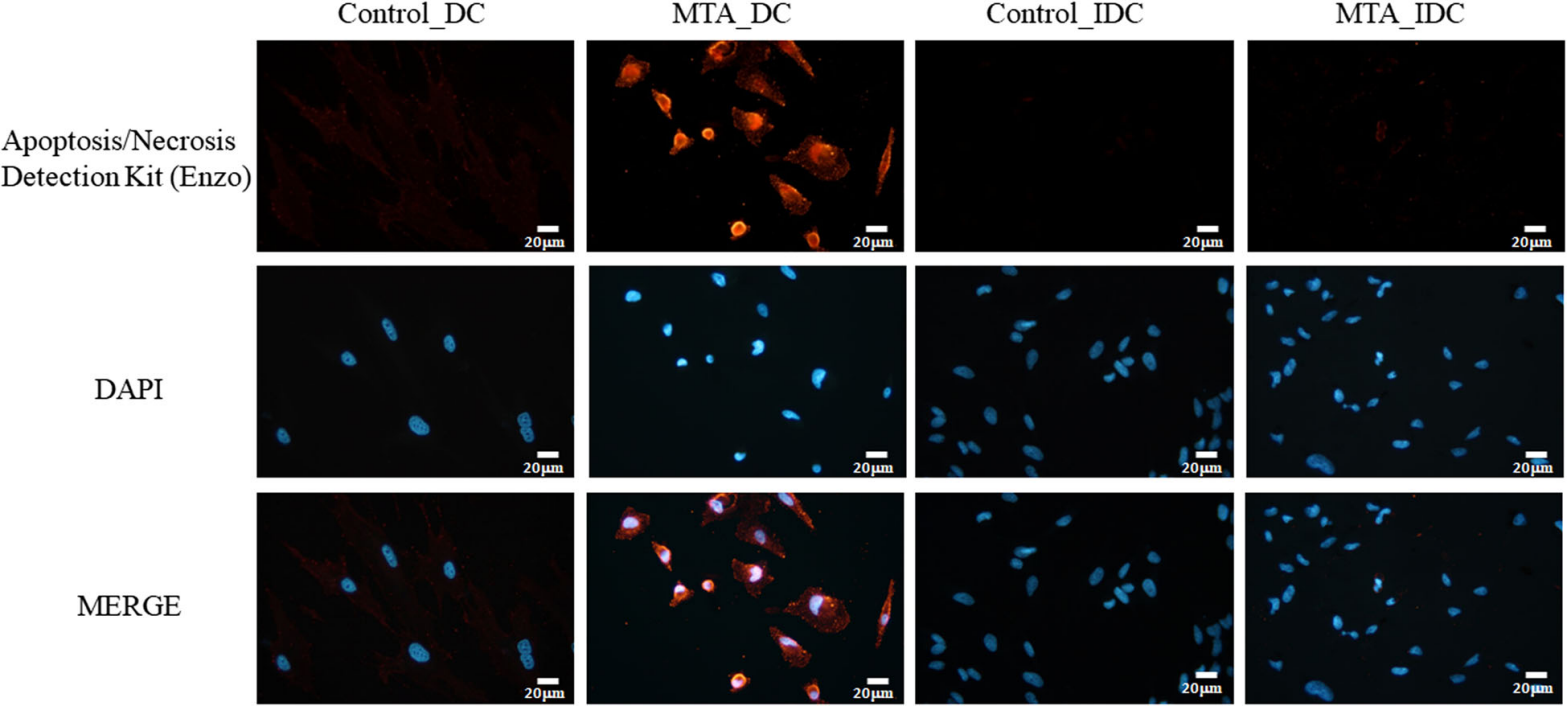
Apoptosis assessment using Annexin V/7-AAD staining Fluorescent microscopic analysis of SHED cells stained with Annexin V/7-AAD. Yellow and red indicates early and late apoptotic cells, respectively. Shown are representative images of three independent experiments. SHED cells in direct contact with MTA for 2 days were positively stained by Annexin V. White bar, 20µm

Fluorescence imaging was conducted to visualize the difference between apoptosis induced and necrotic cell death. After incubation of SHED cells with MTA for 2 days, the number of cells in MTA groups that remained as an adherent monolayer was greatly decreased compared to the control. In addition, floating cells showed morphological changes, with characteristics similar to apoptosis or necrosis. We co-incubated cells with Annexin V EnzoGold (enhanced cyanine), an early marker of phosphatidylserine externalization at the cell membrane, and red emitting dye 7-AAD, a marker of late apoptosis or necrosis at the nucleus. SHED cells in direct contact with MTA for 2 days were positively stained by Annexin V (Fig. 5). The results suggested that direct contact with MTA induced marked early apoptosis in SHED cells.

### MTA-triggered apoptosis involves Bcl-2 and Bcl-xL decreasing

**Fig. 6:**
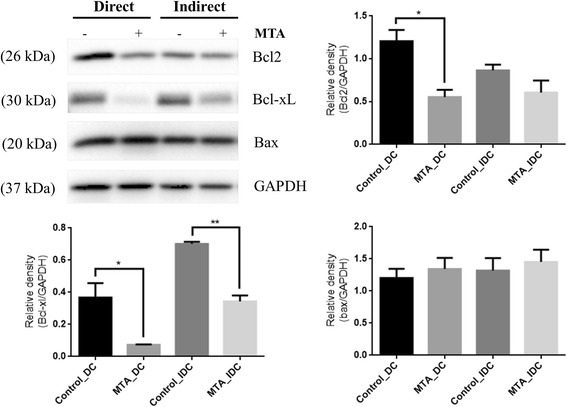
The Bcl-2 family of proteins consists of members that either promote or inhibit apoptosis, and control apoptosis by governing mitochondrial outer membrane permeabilization. The Bcl-2, Bcl-xL and Bax activities were examined using a Western blot assay. The results demonstrated that anti-apoptotic proteins (Bcl-2, Bcl-xL) were significantly decreased compared to the control following 2 days of direct contact with MTA. When the SHEDs were incubated in the environment with the existence of MTA (MTA_IDC), the anti-apoptotic protein BcL-xL was significantly decreased. There was no significant difference between groups in the amount of pro-apoptotic protein (Bax)

To determine whether Bcl-2 protein families are majorly involved in MTA-induced apoptotic cell death, the Bcl-2, Bcl-xL and Bax activities were examined using a Western blot assay. Our results demonstrated that anti-apoptotic proteins (Bcl-2, Bcl-xL) were decreased significantly compared with the control following 2 days of exposure to MTA. Although the pro-apoptotic protein (Bax) was slightly increased within MTA groups, there was no significant difference when compared with the control (Fig. 6).

## Discussion

To mimic the clinical application of tamping material on the dental pulp tissue, the present study designed an environment in which cells were challenged by pressure and MTA-contact. The study used the novel method that can check the attached cells’ reaction after direct contact with MTA. This study showed that 1 week post-set MTA impaired cell viability of SHEDs and the effect of direct contact was more severe. Cell apoptosis was noted especially when SHEDs were in direct contact with MTA.

The present study revealed that post-set MTA inhibits viability of SHEDs in the culture of the first 3 days. It seems that the results from the present in vitro assay challenge the biocompatible image of MTA. Actually, cell culture studies on MTA show that the cell response to the material depends on many factors such as the cell types and the choice of study duration, use of a fresh or cured material, frequency of changing the medium, the use of direct contact or extracts of MTA, and the concentration of the material in the cell culture media [12]. Conclusions drawn from in previous vitro studies are limited due to the use of monolayer cultures exposed to the eluate of the test materials [24].

In clinical practice, fresh mixed MTA is directly applied to the surface of dental pulp and sets thereafter. Set MTA contains calcium hydroxide in a silicate matrix; the presence of calcium hydroxide is what attributes the high pH to MTA [41]. The pH value of MTA is 10.2 after mixing. This value rises to 12.5 at 3 h [3] MTA kept its high pH value that ranged between 11.00 and 12.00 throughout the course of a long-term (78 day) study [10]. MTA acts as a “calcium hydroxide-releasing material” [8], but it does not cause as much caustic injury as pure calcium hydroxide when in contact with tissue [42, 43]. Investigations have shown that it can conduct and induct hard tissue formation [21]. Previously, pure calcium hydroxide itself has been used in pulp therapies for decades [44, 45]. Due to its alkalinity, it induces a superficial necrosis and a moderate inflammatory response in adjacent pulp tissue. Healing is initiated by the resolution of inflammation, recruitment, and differentiation of stem cells, and is completed with the formation of tertiary dentin at the cell-material interface [24, 46]. The formation of reparative dentine in response to calcium hydroxide may not be due to the bioinductive capacity of the material, and instead due to the result of a defense mechanism by the pulp induced by the irritant nature of calcium hydroxide [7, 47]. If one of the successful mechanisms of MTA is the steady releasing of calcium hydroxide, we think that the pulp reaction to the MTA dressing might be a reparative reaction during the healing process.

According to an experiment of cell and tissue reactions to mineral trioxide aggregate, Saidon et al. found that freshly mixed ProRoot MTA caused denaturation of adjacent cells and medium proteins because of the high surface pH. As the materials set with the medium changed every day, the pH changes and the cell injuries subside [48]. In contrast, tissue reaction to post-set ProRoot MTA was well tolerated with a minimal inflammatory response inside the bone [48]. From the above, it suggested that fresh mixed MTA might cause serious cell damage in the soft tissue rather than hard tissue. In another in vitro study comparing the cytotoxicity of Biodentine, MTA Angelus, TheraCal LC and IRM, the researchers found that cell viability was significantly affected and decreased in the presence of materials (*P* < 0.001) in the first 2 cycle periods. At the same time, a significant decrease in the percentage of healthy, non-apoptotic, and non-necrotic cells was detected among the hDPSCs exposed to the above materials (*P* < 0.001). The cytotoxic effects of these materials on hDPSCs were noted [22]. Their direct evaluation method is similar to our indirect contact method. The inhibition in cell growth after stimulation with MTA could be caused by the release of calcium hydroxide from the material and the induced increase in pH [49]. This condition has also been shown with similar materials such as Biodentine [50].

The Alpha MEM, the base of the culture medium, contains 2.2 g/L sodium bicarbonate (NaHCO_3_), 140 mg/L Sodium Phosphate monobasic (NaH_2_PO_4_-H_2_O). It is optimized for 5% CO_2_ in the incubator for proper buffering. We checked the pH of the culture medium changed after being placed onto the MTA. Out of incubator and in absence of CO_2_, the pH of culture medium is 7.8. After MTA was in the culture medium for 3 days, the pH elevated to 8.4. When we cultured SHEDs without MTA for 3 days, the pH was about 8.09. After cultured SHEDs were with MTA for 3 days, the pH was about 8.35. As for the concentration of calcium ions, it was 9.1, > 15, 9.5 and 7.5 mg/dL in culture medium only, culture medium with MTA, SHEDs in culture medium, and SHEDs in culture medium with MTA. The change of pH and concentration of calcium ion in the study is interesting. SHEDs Culturing would mildly lower the high pH and the concentration of calcium ions that related to MTA existing. Besides the bicarbonate and the phosphate system, the culture medium is also composed of 15% FBS that may provide protein buffer system. Although the exact pH level should be lower in the incubator with 5% CO_2_, the buffer system in the culture medium could not stabilize the pH when MTA was existed in this study. The strength of buffers is not exactly as the same as the serum. Further associated study is warranted.

The clinically high success rate for MTA pulp capping or pulpotomy may be due to tissue homeostasis in vivo, and is likewise able to reduce the initially high concentrations of calcium hydroxide and thus alkalinity [8, 51]. This is essential to overcome the dose-dependent toxic effect of calcium silicate-based materials on dental pulp stem cells [52]. A dose effect of MTA on cell viability has previously investigated that concentrations larger than 2 mg/ml of MTA extract were found to have a toxic effect on cells. However, when MTA was prepared at concentrations ranging from 0.1 to 2 mg/ml by a leaching method, the dental pulp stem cells’ (DPSCs) viability increased and DPSCs were able to differentiate into odontoblasts [36]. In clinical practice, the application of fresh mixed MTA into pulp tissue might induce more severe tissue damage in superficial pulp and less in the deeper tissue according to the gradient concentration of calcium hydroxide from MTA.

One study focused on the differentiation of human dental pulp cells (DPCs) after direct contact with MTA. Their results showed that MTA does not influence cell proliferation, but direct contact with MTA is necessary to help differentiate into odontoblast-like cells [53]. Another study about the effect of MTA on mesenchymal stem cells (MSC) claimed that MTA was able to assist MSC adhesion, growth, and migration. However, their morphologic observation of marked MSC under laser scanning microscopy showed worse adhesion to the MTA compared with the control [54]. In contrast, the results of our study are different. Direct contact with MTA significantly decreased cell viability when compared to groups without direct contact. Tunnel and Annexin V/7-AAD staining assay proved that the lower cell viability is due to apoptosis of the cells. More studies including in vivo studies are warranted.

The present study confirms the positive stain of apoptosis cells after direct contact with MTA. In a previous in vitro study about the effect of MTA on pulp cell lines, the authors concluded that MTA induced proliferation, and not apoptosis [6]. The major difference compared to our study is that their indirect contact of MTA with the permeable membrane insert was only for 1 day. The other study showed that when the materials are placed in close proximity to the cells for 3 days, the flow cytometry study for the distribution of vital, early apoptosis, late apoptosis, and necrotic cells revealed that MTA or biodentine treated hDPSCs were significantly less healthy [22]. Collado-Gonzalez, M. et al. tested several materials on SHEDs by their eluates, and the 2 days’ results revealed that MTA and Biodentin did not induce apoptosis [27]. To sum up the results of the above studies, direct contact with MTA may be the key factor for apoptosis of the tested cells.

In the present study, the pressure from the cover slip was also a factor that influences cell viability and proliferation. The pressure from the applicator must be taken into consideration during the studies. However, the control-group setting may control this factor. On the other hand, the pressure did not induce an apoptosis reaction. The shortcoming of this study was that the cell number decreased when in contact with MTA and there was not enough to do a quantitative analysis of the flow cytometry for apoptosis detection. Instead, a qualitative analysis of TUNEL and Annexin V/7-AAD staining was performed in the present study.

## Conclusion

Direct contact with 1-week post-set MTA significantly decreases the viability of SHEDs and induces cell apoptosis. The results suggest that there is a possible cytotoxic effect of pulp tissue when in direct contact with MTA. Further in vivo studies are warranted because different responses would be expected due to the strong alkaline characteristic of fresh mixed MTA.

## Abbreviations

CH: Calcium hydroxide
DAPI: 4′6-diamidino-2-phenylindole
DC: Direct contact
DPSCs: Dental pulp stem cells
FBS: Fetal bovine serum
hDPSCs: Human dental pulp stem cells
IDC: Indirect contact
MSCs: Mesenchymal stem cells
MTA: Mineral trioxide aggregate
SDS: Sodium dodecyl sulfate
SHEDs: Stem Cells from Human Exfoliated Deciduous Teeth
TUMEL: Terminal deoxynucleotidyl transferase (TdT) dUTP nick end labeling
